# Efficacy, Safety and Pharmacoeconomic Analysis of Intravenous Ferric Carboxymaltose in Anemic Hemodialysis Patients Unresponsive to Ferric Gluconate Treatment: A Multicenter Retrospective Study

**DOI:** 10.3390/jcm11185284

**Published:** 2022-09-07

**Authors:** Alberto Rosati, Paolo Conti, Patrizia Berto, Sabrina Molinaro, Federica Baldini, Colin Gerard Egan, Vincenzo Panichi

**Affiliations:** 1 SOC Nefrologia e Dialisi, Ospedale San Giovanni di Dio, 50143 Firenze, Italy; 2 Nephrology and Dialysis Unit, Nephrology Department, Arezzo Hospital, 52100 Arezzo, Italy; 3 Regulatory Pharma Net S.r.L., 56125 Pisa, Italy; 4 National Research Council, Institute of Clinical Physiology (CNR-IFC), 56124 Pisa, Italy; 5 CE Medical Writing S.r.L.S., 56021 Pisa, Italy; 6 Nephrology and Dialysis Unit, Versilia Hospital, 55049 Lido Di Camaiore, Italy

**Keywords:** ferric carboxymaltose, intravenous iron supplementation, iron deficiency anemia, chronic kidney disease, hemodialysis, hemoglobin

## Abstract

Patients undergoing hemodialysis with iron deficiency anemia (IDA) receiving treatment with erythropoiesis-stimulating agents (ESAs) who were intolerant or non-responsive to intravenous (i.v.) ferric gluconate (FG) (hemoglobin; Hb values < 10.5 g/dL or increase in <1 g/dL) or % transferrin saturation; TSAT of <20%) in the previous 6 months were switched to i.v. ferric carboxymaltose (FCM). Changes in iron status parameters, economic and safety measures were also assessed. Seventy-seven hemodialysis patients aged 68 ± 15 years were included. Erythropoietin resistance index decreased from 24.2 ± 14.6 at pre-switch to 20.4 ± 14.6 after 6 months of FCM treatment and Hb levels ≥10.5 g/dL improved from 61% to 75.3% patients (*p* = 0.042). A 1 g/dL increase in Hb levels was also seen in 26% of patients as well as a 37.7% increase in patients achieving >20% increase in TSAT after FCM. Levels of Hb, TSAT and ferritin parameters increased during FCM treatment with a concomitant decrease in ESA. A mixed-model analysis, which also considered gender, confirmed these trends. Safety variables remained stable, no hypersensitivity reaction was recorded and only one patient reported an adverse event after FCM. FCM treatment was associated with a cost saving of 11.11 EUR/patient/month. These results confirm the efficacy, safety and cost-effectiveness of FCM in correcting IDA in hemodialysis patients.

## 1. Introduction

Anemia and iron deficiency are common complications in patients with chronic kidney disease (CKD) and can contribute to the burden of the disease in both end-stage renal disease patients as well as those undergoing dialysis [1,2,3,4]. Anemia and iron deficiency may be associated with symptoms such as fatigue and dyspnoea [5,6] and may increase the risk of mortality and cardiovascular complications [2,7].

Factors contributing to the development of iron deficiency in CKD are reduced dietary iron intake, chronic inflammatory processes, rapid increase in iron requirements during treatment with erythropoiesis stimulating agents (ESA) and, mainly in the case of patients in hemodialysis, chronic blood loss related to treatment. Annual blood loss is approximately 2.5 L for a patient undergoing hemodialysis with a particularly high risk of iron depletion.

Iron deficiency can be defined as absolute or functional [8]; absolute iron deficiency develops when the body’s iron stores are so depleted that there is not enough iron available for hemoglobin production. This is usually indicated by a drop in serum ferritin levels below approximately <15 ng/mL in patients with normal renal function, but occurs at much higher levels in patients undergoing hemodialysis as a result of chronic inflammation and can be associated with elevated levels of C-reactive protein (CRP) [9]. Functional deficit describes the state in which iron cannot be mobilized from stores (despite adequate food intake) to meet the demand for erythropoiesis [8]. Serum ferritin levels may appear normal (200–500 ng /mL) or increased as in chronic inflammatory disorders, whereas transferrin saturation levels (TSAT) will be low (typically <20%).

For patients undergoing hemodialysis, the intravenous route of iron administration is recommended. However, i.v. administration of iron is associated with several limitations, including immunogenic potential, the need to use low doses, a low rate of administration (to prevent acute, iron-induced toxicity and vasoactive reactions), the release of significant doses of extremely reactive and potentially toxic-free iron [10].

Since the advent of intravenous (i.v.) iron complexes, different types of carbohydrates have been selected for these nanomedicines, including dextran, gluconate, sucrose, carboxymaltose, isomalto-oligosaccharide and polyglucose sorbitol carboxymethyl ether [11,12,13].

Carboxymaltose iron [FCM; Ferinject^®^;] is a new generation of iron formulation for parenteral use free of dextran that is designed to overcome the limits of other i.v. iron preparations. The FCM complex is composed of a polynuclear iron hydroxide complexed to carboxymaltose [14]. FCM is a very stable iron complex, can be administered in high doses, releases very low amounts of free iron [15] and appears to be less immunogenic [16].

The availability of adequate doses of iron for erythropoiesis is a crucial element for the response to ESAs. In the case of absolute or functional iron deficiency, higher doses of ESA are required in order to obtain a significant erythropoietic response (i.e., resistance or hyporesponsiveness to ESA). High doses of ESA have been associated with an increased risk of cardiovascular events, therefore, necessitating achievement of adequate TSAT and ferritin levels in patients undergoing dialysis. In a significant proportion of patients, ferric gluconate (FG) still fails to significantly increase available iron even if administered in high doses. Due to its stability and the mechanisms of iron release, FCM may be able to increase the amount of iron available for erythropoiesis, thus reducing the need for ESA [17,18].

Previous studies have shown that FCM is well tolerated and effective in correcting haemoglobin (Hb) levels in non-dialysis [19,20,21,22,23] as well as dialysis patients [24,25,26,27,28]. To date, the majority of studies performed in hemodialysis patients were either limited by the open-label design [24], number of patients included [27,28] or the short follow-up periods [24,25]. 

The aim of our study was to retrospectively evaluate a population of hemodialysis patients in 15 centers across the Tuscany region in Italy in terms of efficacy and safety of the use of FCM in non-responders to FG treatment over a period of 12 months. A pharmacoeconomic evaluation was also performed to determine whether the possible reduction in ESA consumption and other management costs could allow the use of FCM vs. FG to be economically beneficial.

## 2. Materials and Methods

### 2.1. Patients and Study Design

In this retrospective multicentre observational study, patients undergoing hemodialysis treatment were retrospectively evaluated at 15 centers across the Tuscany region who had switched from FG to FCM for at least 6 months due to inadequate response to FG. Patients were enrolled between January 2019 to December 2020, with monthly visits during the pre-switch period (FG treatment) and post-switch period (FCM treatment). Inclusion criteria were the following: (a) hemodialysis patients with anemia treated with ESA and absolute or functional iron deficiency undergoing hemodialysis treatment two or three times/week for at least 12 months, (b) switch to FCM after at least six months of treatment with FG due to ineffective response, in terms of increase in Hb (Hb < 10.5 g/dL or increase <1 g/dL) or TSAT of <20% and (c) an observation period after the switch of at least 6 months. Exclusion criteria were: (a) patients who develop hypersensitivity reactions to FG or FCM during the observation period will not be excluded but will be evaluated for safety only, (b) acute inflammatory status such as rheumatological, autoimmune and/or acute infection, (c) active infections or neoplasms, (d) patients who have undergone blood transfusions during the observation period and (e) pregnancy. Other exclusion criteria were hospitalization during the 6 months prior to enrollment and hospitalization during the study period. The study (protocol number: FCMHD-ID 14157, int. 64/18FI) study was approved by the Regional Ethics Committee for Clinical Studies from the Tuscany Region (Area Vasta della Regione Toscana) on 22 July 2019. Local ethics committee approval from all participating centres and written informed consent for the anonymous use of personal data was also obtained from every patient, in compliance with Legislative Decree no. 211 (24 June 2003). This study complies with the ethical standards laid down in the 1975 Declaration of Helsinki.

### 2.2. Efficacy and Safety Measures

The primary efficacy outcome of this retrospective observational study was to evaluate the reduction in ESA consumption in the sixth months after switching to FCM in patients who did not have a satisfactory response to FG, assessed using the ESA resistance index (ERI) (IU/kg/week/g Hb) [29]. Secondary aims included the evaluation of the reduction in ESA consumption in the third month after switching to FCM in patients, again assessed using ERI. We also evaluated the proportion of patients achieving >1 g/dL increase in Hb and/or TSAT >20% after switching to FCM at 3 and 6 months as well as the proportion of patients achieving hemoglobin levels of ≥10.5 g/dL. In addition to evaluating the efficacy of FCM, we also evaluated the safety of FCM by monitoring vital signs (blood pressure and biochemical variables) reaction to i.v. infusion, hypersensitivity and the presence of any adverse events (AEs). 

### 2.3. Pharmacoeconomic Analysis

Ancillary analysis was performed to evaluate the pharmacoeconomic impact of switching from FG to FCM. In the present study, visits, tests and admissions were established *a priori* according to protocol; therefore, the economic analysis was restricted to drug use only. In addition, patients who were transfused during the study were excluded from the final analysis as transfusions could represent a confounding factor on the clinical outcome measures, hemoglobin and ERI.

Therefore, in the present analysis, data referring to each monthly visit during the study from time −6 months to time +6 months were extracted and tabulated as previously described [27,30] for each patient for the following variables:

(a) ESA consumption, identified as a prescription by the Centre at each visit, for the subsequent one-month period, detected with the variables ESA type (0 = no, 1 = binocrit-alpha-epoetin alpha-erythropoietin alpha, 2 = epoetin zeta-retacrit-zeta-retacrit, 3 = darbopoetin-aranesp) and prescribed as a weekly dose in IU (0 = no);
(b) Iron consumption, identified as a prescription by the Centre at each visit, for the subsequent one-month period, detected with the variables Iron type (0 = no, 1 = FCM, 2 = gluconate, 3 = saccharate) and prescribed as a monthly dose in mg (0 = no).

After visual inspection of resource-use data, missing data for ESA and iron prescription was found for some patients, so the following actions were taken: for patients with at least 3 measurements available with indication of the ‘type’ and ‘dose’ of ESA or iron, in the absence of specification of the ‘type’, but in the presence of the prescribed ‘dose’, the ‘type’ indicated at the last available measurement was used.

Nineteen patients (24.7%) for which the number of prescriptions was too small to allow any quantification were excluded from the economic evaluation. In order to attribute the correct economic value to the resources ESA consumption and iron consumption, the prices for the treatments under analysis were extracted from the AIFA database (https://www.aifa.gov.it/liste-farmaci-a-h; accessed on 21 February 2022), and the relative values per IU or mg were obtained and applied to each patient’s monthly consumption.

Consumption of ESA and iron on the cohort was calculated as the average consumption per patient in the pre-switch (time −6 to switch) compared to the post-switch (time +1 to time +6). These mean values were multiplied by the relative cost per IU (in the case of ESA) and per mg (in the case of iron), considering the various treatment options provided (for ESA: EPO-alpha, EPO-zeta, darbepoetin; for Iron: FG, FCM) at their respective prices. The mean pre/post for the cohort was calculated as the average of the mean values per patient.

### 2.4. Sample Size Calculation

The sample size was calculated to demonstrate that HD patients who tend to be anemic with FG therapy may benefit from FCM treatment in terms of dose reduction of ERI = EPO (IU/week/kg) /Hb (g/dL) (primary endpoint of the study). Based on previous studies [31,32], the mean ERI of the patients in HD was found to be 11.7 (standard deviation, SD of 8.2). Patients proposed to change therapy tend to have higher ERI values than the average of all patients in HD. It was assumed that they have a mean value before displacement at least 0.1 SD higher than the mean (11.7 + 0.10 × 8.2 = 12.5). We also cautiously assumed that there is only a moderate correlation between the pre and post measures (*r* = 0.5) and therefore pre-post SD = √ (SDpre2 + SDpost2−2 × *r* × SDpre × SDpost) is estimated at 8.2 (assuming homogeneity of variance (SDpre = SDpost). To have an 80% probability (power) to detect a statistically significant difference (at the two-tailed alpha level of 0.05) and an absolute ERI change of 2.5 (doses of EPO/g of Hb, corresponding to a percentage reduction of 20%), 87 patients would be needed, corresponding to a standardized effect size of 0.3.

### 2.5. Statistical Analysis

Data are presented as mean and standard deviation for continuous variables and absolute numbers and percentages for categorical variables. Parameters that were not normally distributed were also presented as median and lower and upper 95% confidence intervals (95% CIs). Comparisons in differences in variables between two time points (two samples) were performed by the Wilcoxon test. The change in outcome measures (ERI, TSAT, ferritin levels) following switch from FG to FCM were assessed using mixed analysis models to compare the following 4 time points; 6 months prior to switch, 3 months pre-switch, 3 months post-FCM switch (+3 months) and 6 months post-FCM switch (+6 months) to baseline values (when switch was undertaken from FG to FCM). The effect of switching on dose of iron/month was also assessed by comparing the first time point to each subsequent time point separately for pre- and post-FCM switch. Other variables tested in the different mixed models included smoking status, presence of diabetes, antihypertensive therapy and dialysis type. SPSS Software (version 26, IBM Corp, Chicago, IL, USA) was used to perform descriptive analyses and STATA (version 16, StataCorp, College Station, TX, USA) for mixed model analysis. All quoted *p*-values are two-tailed and a *p*-value ≤ 0.05 was considered statistically significant. 

## 3. Results

### 3.1. Baseline Clinical Characteristics

In this retrospective observational study, a total of 77 patients undergoing hemodialysis were switched to FCM (Ferinject^®^, Vifor International, St Gallen, Switzerland) following lack of efficacy of FG in the previous 6 months. Baseline clinical characteristics of patients in the visit prior to switching to FCM are summarised in Table 1. The majority were male (64.9%) aged 68 ± 15 years. Comorbid diseases included diabetes mellitus (*n* = 19; 24.7%), obesity (*n* = 14; 18.2% and the most common primary renal disease was glomerulonephritis (*n* = 18; 23.4%) and vascular disease (*n* = 18; 23.4%), followed by diabetic nephropathy (*n* = 16; 20.8%). Just under half of patients were undergoing hemodialysis (*n* = 37; 48.1%), followed by online hemodiafiltration (*n* = 26; 33.8%), and almost three-quarters of patients were previously receiving anti-hypertensive therapy (*n* = 57; 74%). At baseline, mean hemoglobin levels were 10.3 ± 1.1 g/dL and 61% (*n* = 47) of patients had Hb levels ≥10.5 g/dL. TSAT levels were 15.2 ± 5.8% and mean weekly dose of ESAs were 15,434 ± 10,307 IU and ferritin levels were 179.4 ± 234.7 ng/mL.

### 3.2. ESA and Iron Treatment over the Study Period

**Figure 1:**
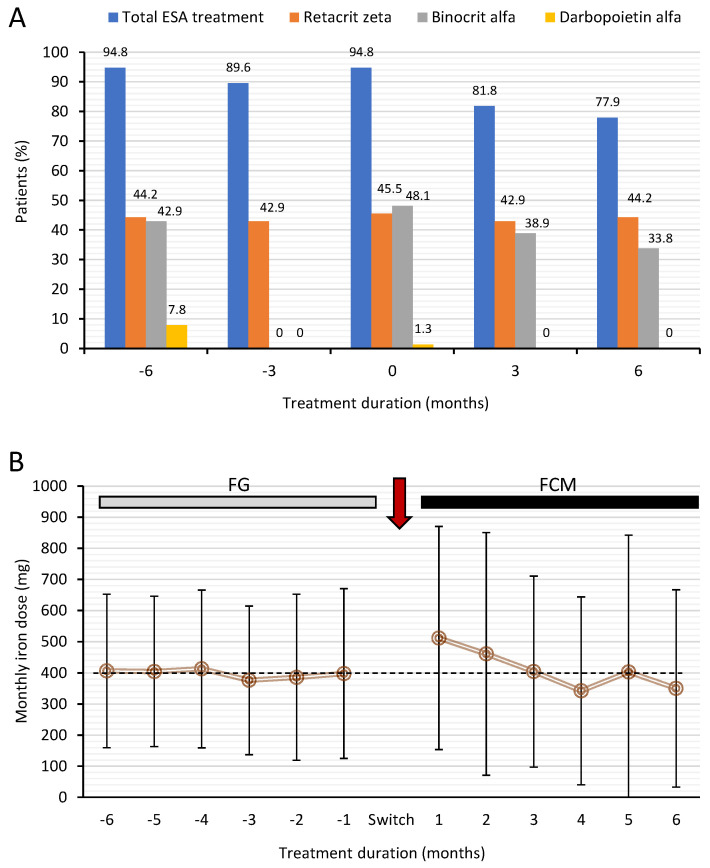
Treatment with ESA and iron in hemodialysis patients who switched from FG to FCM. (**A**), the proportion of patients (%) treated with different ESAs during FG and FCM treatments over the 12 months; (**B**), monthly iron dose in patients during FG and FCM treatments over the 12 months. The dotted line represents iron dose at the pre-switch visit (−1 months). Monthly iron dose data are presented as mean ± SD whereas. FCM = ferric carboxymaltose, FG = ferric gluconate.

The proportion of patients receiving ESA or iron treatment over the study period is shown in Figure 1. The majority of patients (~80%) were receiving binocrit alfa or retacrit zeta in similar proportions at the first visit (−6 months) during FG treatment and the proportion of patients receiving binocrit alfa was observed to decrease following switch from FG to FCM (Figure 1A). This change was reflected in the proportion of patients receiving any ESA treatment, where a significant decrease from 94.8% to 77.9% was observed (*p* < 0.001).

During the switch period (FG treatment), mean monthly iron dose remained stable at approximately 400 mg (range 397.9 ± 272.6 to 412.7 ± 253.7 mg) (Figure 1B). When patients were switched to FCM, mean iron dose was 512 ± 358 mg and decreased every month by about 50 mg to 342 ± 302 mg at 4 months and remained stable thereafter. The mean dose for the entire period of FG treatment was 394 ± 203 mg compared to 412 ± 243 mg in the period under FCM treatment with no statistical difference between them (*p* = 0.53).

### 3.3. Efficacy Outcome Measures

**Figure 2:**
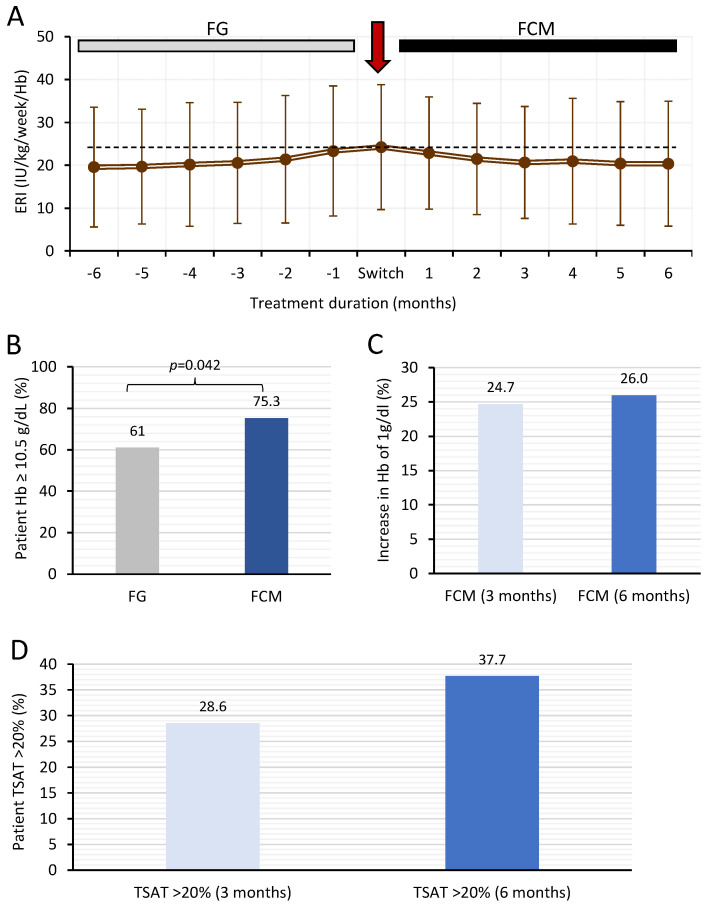
Primary efficacy measures in hemodialysis patients who switched from FG to FCM. (**A**), change in ERI during FG and FCM treatments over the 12 months. (**B**), the proportion of patients (%) achieving hemoglobin levels ≥10.5 g/dL during FG and FCM treatments; (**C**), the proportion of patients (%) showing a 1 g/dL increase in hemoglobin levels at 3 and 6 months of FCM treatment; (**D**), the proportion of patients (%) achieving levels of TSAT >20% at 3 and 6 months of FCM treatment. ERI data are presented as mean ± SD whereas all other data are presented as %. Dotted line represents ERI at the switch visit. ERI = erythropoietin resistance index, FCM = ferric carboxymaltose, FG = ferric gluconate, TSAT = transferrin saturation.

The primary objective of this study was to evaluate the reduction in ESA consumption at 3- and 6-months following switch to FCM, assessed using the ERI. Mean ERI values increased from 19.6 ± 14 at −6 months to 24.2 ± 14.6 prior to switching to FCM, equating to a 23.8% increase (Figure 2A). In contrast, switching to FCM resulted in a time-dependent decrease in ERI values, from 24.2 ± 14.6 to 20.7 ± 13.1 and 20.4 ± 14.6 at 3 and 6 months respectively, equating to a 16% decrease at 6 months. 

The proportion of patient achieving a hemoglobin level of ≥10.5 mg/dL increased from 61% to 75.3% (*p* = 0.042) in the pre and post-FCM phase, respectively (Figure 2B). Furthermore, the proportion of patients showing a 1 g/dL increase in hemoglobin levels of 24.7% and 26% at 3 and 6 months, respectively, with FCM compared to treatment with FG (pre-switch visit) (Figure 2C). Achievement of TSAT >20% was observed in 28.6% of patients at 3 months and 37.7% at 6 months following the switch to FCM compared to pre-switch (Figure 2D).

**Figure 3:**
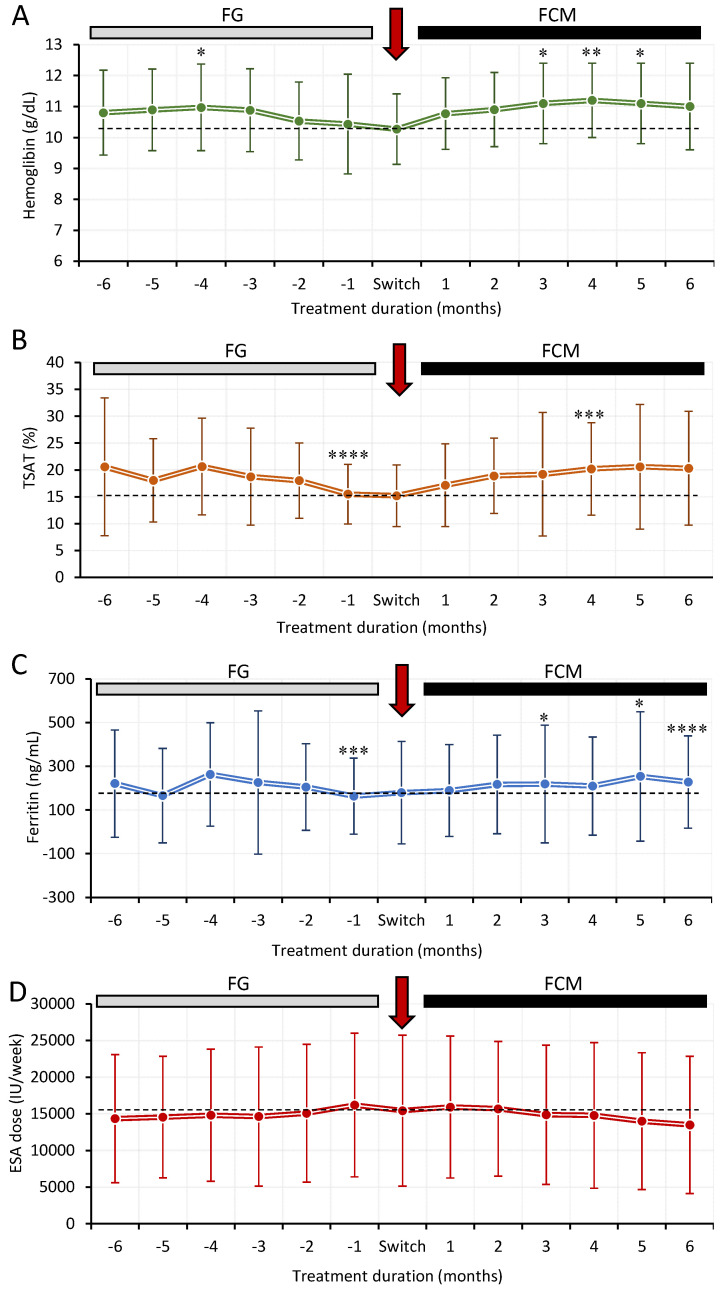
Secondary efficacy measures in hemodialysis patients who switched from FG to FCM. (**A**), change in haemoglobin levels during FG and FCM treatments over the 12 months. (**B**), change in TSAT levels during FG and FCM treatments over the 12 months. (**C**), change in ferritin levels during FG and FCM treatments over the 12 months. (**D**), change in ESA dose during FG and FCM treatments over the 12 months. Dotted line represents levels of variables at the switch visit. Data are presented as mean ± SD. *p*-values denote statistical significance for FG treatment (vs. −6 months) or FCM treatment (vs. switch visit) where * = <0.05, ** = <0.01, *** = <0.001 and **** = <0.0001. ESA = erythropoietin stimulating agent, TSAT = transferrin saturation.

Secondary efficacy outcome measures included the evaluation of changes in hemoglobin, TSAT, ferritin and ESA dose at each visit during FG and after the switch to FCM and are presented in Figure 2. Although a significant increase was observed in hemoglobin levels from −6 months to −4 months during FG treatment (10.8 ± 1.4 g/dL to 11 ± 1.4 g/dL, *p* < 0.05), levels decreased in subsequent visits up to the switch to FCM (Figure 3A). Following the switch to FCM (10.3 ± 1.1 g/dL), hemoglobin levels increased (significantly different at 3 (11.1 ± 1.3 g/dL), 4 (11.2 ± 1.2 g/dL) and 5 (11.1 ± 1.3 g/dL) months vs. pre-switch levels) in a time-dependent manner over the 6-month treatment period, with levels of 11 ± 1.4 g/dL achieved at 6 months. Similar to hemoglobin levels, TSAT levels also decreased from 20.6 ± 12.9% at −6 months to 15.2 ± 5.8% pre-switch (*p* < 0.0001 vs. −6 months) and increased each month following switch to FCM reaching statistical significance at 4 months vs. pre-switch levels (15.2 ± 5.8% vs. 20.2 ± 8.6%, *p* < 0.001) (Figure 3B). Despite the large variation in values, mean ferritin levels were observed to decrease significantly at −1 month (163.4 ± 174.2 ng/mL) vs. −6 months (220.7 ± 245.9 ng/mL) (FG treatment) with significant increases observed at 3 (219.1 ± 269.2 ng/mL), 5 (253.7 ± 296.1 ng/mL) and 6 months 228.2 ± 211.1 ng/mL) after the switch to FCM vs. pre-switch levels (179.4 ± 234.7 ng/mL) (Figure 3C). Mean ESA dose was observed to increase gradually during the 6 months with FG treatment (from 14,338 ± 8755 IU at −6 months to 15,434 ± 10,307 at pre-switch visit) and decreased over time following FCM treatment (13,486 ± 9357 IU at 6 months) (Figure 3D).

### 3.4. Mixed Model Analysis

Using different mixed models, we next wanted to explore the effect of switching from FG to FCM on the following variables: ERI, TSAT, ferritin levels and iron monthly dose. For ERI, TSAT and ferritin levels, the time of the switch from FG to FCM was used as a baseline comparison for the time points pre-switch, −6 and −3 months as well as time-points post-switch, +3 and +6 months. For monthly iron doses, it was not possible to compare pre- and post-FCM switch values to the time of switch. Therefore, pre-switch iron dose values were compared to the first pre-switch time (−6 months), and the post-switch iron dose values compared to the first post-switch time (+1 month). In all models, gender was also considered. 

Results derived from mixed model analysis are shown in Table 2. Whereas female gender increased ERI by 9.96 (compared to male patients), all values were found to be statistically significant compared to values when the switch from FG to FCM was undertaken. ERI values tend to increase (from −6 to −3 months) whereas the opposite was observed during the post-FCM switch phase (i.e., FCM decreased ERI). Using the same approach, we evaluated the effect of switching on TSAT values. No effect from gender was observed and TSAT values decreased from −6 to −3 months whereas an increase was observed at +3 and +6 months vs. switch values. Ferritin levels were not affected by gender and tended to decrease in the pre-switch phase (although not statistically significant) and increased in the FCM phase, attaining statistical significance at +6 months (+5.45%, *p* < 0.0001). Although no significant difference in pre-iron monthly doses were observed, the majority of iron dose values significantly decreased after FCM treatment compared to pre-FCM doses. Additional variables tested using mixed model analysis included smoking status, presence of diabetes, antihypertensive use and type of dialysis, and these variables did not reveal any statistically significant difference pre- or post- switch (data not shown).

### 3.5. Economic Analysis

Pharmacoeconomic analysis showed that switching to FCM compared to FG was associated with a cost saving of EUR 11.11 per patient per month, due to the reduction in costs of ESA treatment (Table 3).

### 3.6. Safety Assessment

A summary of blood pressure and clinical biochemical parameters in the pre- and post-switch phase of the study are presented in Table 4. BP did not change after the switch from FG to FCM (median SBP; 131.5 mmHg and DBP; 71.5 mmHg) before FCM and 130 mmHg for SBP and 70 mmHg for DBP after FCM. Serum calcium and phosphorus levels increased slightly following the switch to FCM (reaching statistical significance for calcium levels at 6 months vs. pre-switch, *p* < 0.05) but these changes were not considered clinically relevant. Parathyroid hormone levels remained stable over the study period with a significant decrease observed after 6 months (292.5 pg/mL vs. 209 pg/mL, *p* < 0.05). All other biochemical and vital signs remained stable over the study period. No hypersensitivity reaction was recorded during FCM infusions over the observation period (Table 5) and only one patient reported an adverse event (gastralgia) following the switch to FCM at 3 months compared to a total of five adverse events observed when patients were receiving FG (Table 5).

## 4. Discussion

The main findings from this multicentre retrospective observational study show that in anemic hemodialysis patients unresponsive to FG treatment, FCM was effective in correcting anemia and reducing ESA treatment. Although ERI increased in the period during FG treatment, a decrease was observed after switching to FCM. Furthermore, the percentage of patients achieving target Hb levels of ≥10.5 g/dL improved from 61% during FG treatment to 75.3% after FCM treatment and the proportion of patients showing a >20% increase in TSAT was 37.7% at 6 months. Whereas mean levels of Hb, TSAT and ferritin decreased over the 6 months during FG treatment, levels of these same parameters increased following the switch to FCM with a concomitant decrease in ESA dose. Mixed model analysis, which also considered gender, confirmed these trends. Over the 12-month observation period, biochemical parameters and vital signs remained stable, no hypersensitivity reaction was recorded and only one patient reported an adverse event following the switch to FCM. Economic analysis revealed that FCM compared to an FG-based scenario was associated with a cost saving of EUR 11.11 per patient per month, due to the reduction in costs of ESA treatment.

Our results corroborate with findings from previous studies in patients undergoing dialysis that have shown that FCM is effective in correcting haemoglobin (Hb) as well as having a favourable tolerability profile [24,25,26,27,28]. 

An earlier study in 2010, using a different FCM formulation by Covic et al., evaluated the efficacy and safety of i.v. FCM in 163 patients with IDA undergoing haemodialysis [24]. The primary objective of their trial was to assesses the safety of FCM during the 4-week treatment period. A total of 193 AEs was reported in 89 (54.6%) patients and serious AEs occurred in 12 out of 163 (7.4%) patients, but none of these was related to FCM. Almost three-quarters of patients (73.6%) received ESAs, but the dose remained stable during the study. Mean haemoglobin levels increased from 9.1 ± 1.30 g/dL at baseline to 10.3 ± 1.63 g/dL at follow-up as well as an improvement in TSAT (17.4% to 31%) and ferritin levels (67.3 to 33.6 µg/l). In terms of presence of AEs, we observed only one AE (1.3%) over the 6-month treatment period with FCM, mean haemoglobin levels increased from 10.3 ± 1.1 to 10.9 ± 1.3 g/dL and ferritin levels increased from 179.4 ± 234.7 to 189.2 ± 209.9 ng/mL after 4 weeks. Patients were considerably older in our study (68 ± 15 vs. 44.9 ± 12.7 years) but had less severe anemia prior to initiating FCM treatment. 

A randomised multi-center study by Charytan evaluated the efficacy of i.v. FCM vs. standard medical care (SMC) in haemodialysis as well as non-hemodialysis patients [25]. The incidence of AEs was similar between hemodialysis groups: 42% in the FCM group (*n* = 50) and 40.4% (*n* = 47) in the SMC group. No differences were observed in the proportion of patients achieving target haemoglobin levels between FCM and SMC groups.

Hofman and colleagues showed that in 221 stable hemodialysis patients, switching from iron sucrose to FCM was associated with a significant improvement in iron status and hemoglobin values, unrelated to i.v. iron dose [26]. The follow-up period post FCM switch was 9 months. Hemoglobin levels over the 9 months increased in all groups whereas the weekly iron dose was significantly lower when patients received FCM compared to iron sucrose (48 vs. 55 mg/week, *p* = 0.04). Furthermore, serum ferritin and TSAT increased in all groups as well as a decrease in weekly doses of ESA was observed after switching to FCM [26]. 

Similar to this study, we also observed an improvement in haemoglobin values, albeit to a lower extent (from 10.3 ± 1.63 g/dL to 11 ± 1.4 g/dL at 6 months) with a similar improvement in ferritin and TSAT values that were independent of any increase in mean iron dose. Hofman observed an improvement in iron status parameters despite a decrease in iron dose during FCM treatment vs. iron sucrose treatment [26]. A potential weakness of their study was that they did not assess the tolerability or safety of either iron preparation, although other previous studies have not observed any discernible difference in these preparation in terms of safety [33]. 

Recently, Lacquaniti et al. retrospectively evaluated the efficacy of FCM in 25 patients previously treated with FG [28]. Over the 4-year observation period, lower doses of EPO were administered during FCM treatment compared to during FG. Furthermore, stable and on target levels of hemoglobin were maintained with better control of anemia through high levels of ferritin and TSAT. FCM increased TSAT levels by 11.9% (*p* < 0.001) compared to FG. The proportion of patients with TSAT < 20% were reduced during FCM. The monthly EPO dose was reduced in the FCM period (−6,404.1 IU, *p* = 0.003), as well as the ERI (*p* = 0.004). During the period with FCM, ferritin levels were higher than during FG (*p* < 0.001). We also observed a similar improvement across iron status parameters although the increase in some parameters (e.g., TSAT) may be attributed to the slightly longer follow-up period (9 months). Our study also benefitted from a more robust sample size (*n* = 77) compared to that of Lacquaniti et al. (*n* = 25) [28].

The PIVOTAL study compared a proactive versus a reactive strategy with i.v. iron sucrose on 2300 HD patients and reported a lower risk for a composite of events in the high-dose i.v. iron group, with better control of anaemia and iron, and a similar safety profile [34]. In our study, patients received a mean cumulative iron dose of about 4800 mg after 12 months (2346 mg in the FG period and 2484 mg in the FCM period), which is higher than that in the proactive group of PIVOTAL study (median 3200 mg/year). Because of the different study design and study population, our results cannot be directly compared with those of the PIVOTAL study. 

A similar study in terms of design by Rognoni et al., carried out on 38 adult dialysis patients with IDA followed at the Verona Nephrology Unit in Italy, confirms the efficacy of FCM vs. FG over 12 months [27]. Although limited by the lower sample size (*n* = 38), the proportion of patients who achieved haemoglobin levels ≥10.5 g/dL increased from 66 to 82% (61 to 75.3% in our study) and the proportion of patients with TSAT >20% increased from 0 to 60% after switching to FCM (0 to 37.7% in our study), with similar improvement in ferritin and ERI.

It is worth noting that although low values of ferritin and TSAT were observed during the FG treatment period, the (mean) monthly iron dose remained the same. We cannot offer a clear explanation for this. This was a retrospective observational study and these findings reflect the current practice in centers in the Tuscany region of Italy. We believe that the management of anemia can indeed vary between centers where individual hospital policies/practice are adopted. This was an observational study and our real-life analysis added a unique characteristic in this regard. One of the key values of this study is that it helps to identify areas of improvement in everyday clinical practice. A lack of uniformity across different centers warrants further investigation.

Results of our economic analysis corroborate with findings from other published Italian economic studies. The study by Rognoni et al. also included an economic component and they showed that switching from FG to FCM allowed a cost reduction per patient/month in the range 14–46 EUR, considering, respectively, the use of biosimilar ESA or originator ESA [27]. In another study, based on the adaptation to the Italian environment of a Dutch real-world study in dialysed patients [26], Aiello et al. concluded that “the switch to FCM results in savings of −12.47 EUR/patient/week (−21%) in all patients, with even greater savings in the subgroup of iron-deficient patients −17.28 EUR (−27%) and in anaemic patients −23.08 EUR (−32%)” [30]. More recently, Minutolo et al. reported data from a single-centre study on the use of FCM in 59 non-dialysis CKD patients with iron-deficiency anaemia, concluding that compared to FG, FCM is associated with a reduction in ESA doses during the study (−26%) with a saving in ESA expenditure of 211/patient over 6 months, more than enough to offset the higher cost of FCM vs. FG (average FCM consumption 847 + 428 mg over 24 weeks) [20]. 

The difference in average cost per patient/month calculated in the present study, corresponded on average to −11.11 EUR/patient is represented by an important reduction in the monthly cost of ESA, as explained by the higher average consumption in IU in the post-switch phase of the study. Therefore, the higher cost of iron appears to be more than compensated by the saving in ESA consumption over the entire study period.

Several potential mechanisms to explain why FCM may be more effective than iron sucrose or FG in replenishing iron stores have been previously proposed [26]. First, FCM is recognized to have increased bioavailability of elemental iron vs. iron sucrose and FCM has a higher molecular weight than iron sucrose (150,000 vs. 43,300 Da) and a longer half-life (7–12 vs. 5–6 h) [35]. Second, FCM is more stable than iron sucrose, which prevents release of labile iron into the blood, where it can saturate transferrin and lead to significant amounts of non-transferrin bound iron [36]. Third, a recent pharmacokinetic study by Rostoker et al. performed on 54 dialysis patients suggests that the benefit afforded by FCM over ferric sucrose appears to be due to an improved availability to bone-marrow with minimal trapping in liver and spleen in contrast to iron sucrose and FG [37].

## 5. Study Limitations

The main limitations of this study lie in the retrospective design and the lack of a control group. However, this was a real-life retrospective analysis performed across 15 dialysis centres in the Tuscany region of Italy and our results may be tentatively extended to other regions across the Italian peninsula. Although a larger sample size would have been desired, we were still able to detect a clear-cut improvement in the same patients in terms of correcting hemoglobin following switching to FCM. The 6-month follow-up period allowed the possibility to also capture important data on potential hypersensitivity reactions or drug-related side effects as well as observe the temporal change in a range of parameters. Moreover, we also observed the same improvement using mixed analysis models, where we also considered a range of confounding variables. For some variables, not all data were available (<5% of cases) as nephrologists did not always collect all variables at each visit for all patients. However, this was observed in both pre- (FG treatment) and post-switch to FCM to the same extent.

Although we comprehensively assessed the efficacy, safety and economic effect of switching from FG to FCM, no data were collected on patient’s perspective on the two treatments in terms of patient reported quality of life measures. 

It is recognized that an economic evaluation should ideally be conducted on the consumption of all healthcare resources generated by the patient. In particular, for a study performed in the context of the healthcare system and consumption of resources, the analysis should focus on direct healthcare costs: visits, drugs, tests and admissions. However, in the present study, visits, tests and admissions were all established *a priori* according to protocol, therefore the economic analysis was restricted to drug use only. Furthermore, nineteen patients (24.7%), for which the number of prescriptions was too small to allow any quantification, were excluded from the economic evaluation.

Since this was a real-world study involving 15 different dialysis centers, no clear defined protocol was followed in terms of iron doses administered. Each dialysis unit had the possibility to follow their individual dosing protocols or regimes in order to achieve haemoglobin target values. Indeed, it is possible that since this study was performed across multiple centers within a single region, there may have been bias in terms of treatment policies that may be regionally specific. In this regard, it should also be noted that different ESA types were also administered. 

A small number of patients (*n* = 3) changed dialysis technique after FG treatment. However, mixed analysis models (which also included dialysis type) did not detect an influence of this parameter on outcome measures.

## 6. Conclusions

The primary objective of this multicentre observational study was to evaluate the reduction in ESA consumption 6 months after switching to FCM in patients who did not have a satisfactory response to FG, as assessed by the ERI. Mixed model analysis revealed that the use of the FCM was more effective than FG, since ERI was observed to increase with FG whereas ERI values tended to decrease in the period following switch to FCM. This analysis also highlighted an increase in TSAT values in the post compared to pre-switch period, as well as a concomitant increase in ferritin values and decrease in monthly iron dose. FCM was also associated with a cost saving of 11.11 EUR/patient/month. Taken together, these results point towards a robust and long-term benefit of FCM over FG in correcting IDA in hemodialysis patients as well as being cost-effective and providing a favourable tolerability profile.

## Figures and Tables

**Table 1:** Baseline clinical characteristics of patients on dialysis.

Clinical Characteristics	*n* = 77
*General*	
Age (years)	68 ± 15
Male gender, *n* (%)	50 (64.9)
Body mass index (kg/m^2^)	25.9 ± 4.9
Smoker, *n* (%)	16 (20.8)
*Comorbid diseases*, *n (%)*	
Diabetes	19 (24.7)
Obesity (BMI ≥30 kg/m^2^)	14 (18.2)
*Aetiology of CKD*, *n (%)*	
Glomerulonephritis	20 (26.0)
Vascular	18 (23.4)
Diabetic nephropathy	16 (20.8)
Family/hereditary	8 (10.4)
Pyelonephritis	3 (3.9)
Secondary nephropathy	2 (2.6)
Congenital	1 (1.3)
Other	9 (11.7)
*Dialysis*, *n (%)*	
Hemodialysis	37 (48.1)
Online hemodiafiltration	26 (33.8)
Hemodiafiltration	12 (15.6)
Acetate-free biofiltration	1 (1.3)
Dialysis adequacy (KT/V)	1.28 ± 0.23
*Previous intervention/treatment*, *n (%)*	
Vascular access; AVF/CVC	65 (84.4)/12 (15.6)
Antihypertensives	57 (74)
Transplant	12 (15.6)
Peritoneal dialysis	9 (11.7)
*Biochemical/laboratory/ESA*	
Hemoglobin (g/dL)	10.3 ± 1.1
Hemoglobin ≥ 10.5 (%)	47 (61)
TSAT (%)	15.2 ± 5.8
Weekly ESA dose (IU)	15,434 ± 10,307
Monthly iron dose (mg)	397.9 ± 272.6
Ferritin (ng/mL)	179.4 ± 234.7
Serum calcium (mg/dL)	8.6 ± 0.5
Serum phosphorus (mg/dL)	5.1 ± 1.6
Parathyroid hormone (pg/mL)	334.9 ± 227.8
C-reactive protein (mg/dL)	1.0 ± 1.2
Albumin (g/dL)	3.97 ± 3.6

AVC = arteriovenous fistula, BMI = body mass index, CVC = central venous catheter, CKD = chronic kidney disease, ESA = erythropoiesis-stimulating agents, TSAT = transferrin saturation. Data presented as mean ± standard deviation or frequencies as number (%). Baseline values were taken at the visit prior to FCM switch.

**Table 2:** Mixed models to explore the association between different variables pre- and post-FCM switch.

Model and Variable	Coefficient	Lower 95% CI	Upper 95% CI	SE	Z-Value	*p*-Value
**Model #1** **ERI Index**						
Gender (female)	9.96	4.54	15.40	2.77	3.60	**<0.0001**
−6 months	−4.11	−6.16	−0.68	1.04	−3.93	**<0.0001**
−3 months	−2.7	−4.75	−0.68	1.03	−2.62	**0.009**
+3 months	−2.19	−4.21	−0.17	1.03	−2.12	**0.034**
+6 months	−3.72	−5.79	−1.64	1.06	−3.50	**<0.0001**
**Model #2** **TSAT (%)**						
Gender (female)	−1.64	−4.69	1.4	1.55	−1.05	0.29
−6 months	5.25	3.34	7.17	0.97	5.39	**<0.0001**
−3 months	1.94	−0.56	3.94	1.02	1.91	0.06
+3 months	4.17	2.2	6.14	1.00	4.16	**<0.0001**
+6 months	5.45	3.52	7.40	0.99	5.51	**<0.0001**
**Model #3** **Ferritin (ng/mL)**						
Gender (female)	−30.17	−128.78	68.43	50.30	−0.60	0.55
−6 months	33.93	−8.92	76.77	21.85	1.55	0.12
−3 months	6.65	−37.01	50.31	22.27	0.30	0.77
+3 months	34.71	−9.23	78.65	22.42	1.55	0.12
+6 months	57.31	13.47	101.15	22.36	2.56	**0.010**
**Model #4** Iron dose (ng/mL) * **pre-switch**						
Gender (female)	14.51	−79.37	108.39	47.89	0.30	0.76
−2 months	−3.15	−58.1	51.79	28.03	−0.11	0.91
−3 months	5.66	−50.69	62.01	28.74	0.20	0.84
−4 months	−22.76	−77.64	32.13	28.00	−0.81	0.42
−5 months	−19.06	−74.17	36.05	28.11	−0.68	0.49
−6 months	−7.11	−61.78	47.56	27.89	−0.25	0.80
**Model #5** Iron dose * post-switch						
Gender (female)	24.71	−86.64	136.06	56.81	0.43	0.66
+2 months	−55.78	−146.68	35.13	46.38	−1.20	0.23
+3 months	−106.18	−199.46	−12.91	47.58	−2.23	**0.026**
+4 months	−183.08	−276.37	−89.79	47.59	−3.85	**<0.0001**
+5 months	−123.73	−216.62	−30.85	47.39	−2.61	**0.009**
+6 months	−165.16	−258.05	−72.28	47.39	−3.49	**<0.0001**

Monthly iron dose in ng/mL. * It was not possible to compare pre- and post-FCM switch values to the time of switch. Therefore, pre-switch iron dose values were compared to the first pre-switch time (−6 months), and the post switch iron dose values with respect to the first post-switch time (+1 month). Statistically significant *p*-values are represented by bold text. CI = confidence interval, FCM = ferric carboxymaltose, SE = standard error.

**Table 3:** Economic analysis over the 12-month study period (*n* = 58).

	Use of Resources			Cost/Patient/Month		
	ESA	ESA	Iron	ESA	Iron	Total
	IU/Week	IU/Month	mg/Month	Cost (EUR)	Cost (EUR)	Cost (EUR)
Pre-switch	14.8	64.27	420	100.62	3.66	104.28
Post-switch	15.15	65.79	340	66.85	26.32	93.17
Difference	350.9	1523.9	−80.02	−33.77	22.67	−11.11

ESA = erythropoietin-stimulating agent, IU = international unit.

**Table 4:** Mean levels of arterial blood pressure, biochemical variables and dialytic variables at 3-month intervals with FG and after FCM treatments.

	Follow-Up Period (Months)				
Characteristic, Median (95% CI)	−6	−3 ^§^	0 (Switch)	+3	+6
SBP (mmHg)	135 (130–140)		131.5 (130.0–140.0)	135 (130–140)	130 (130–140)
DBP (mmHg)	72 (70–80)		71.5 (70.0–80.0)	76 (70–81)	70 (70–80)
Serum calcium (mg/dL)	8.60 (8.50–8.90)		8.60 (8.40–8.80)	8.7 (8.7–9.1) *	8.8 (8.6–9.1) *
Serum phosphorus (mg/dL)	5.20 (4.90–5.70)		4.98 (4.57–5.30)	5.30 (5.0–5.60)	5.5 (5.2–6.1)
Parathyroid hormone (pg/mL)	221 (178–261)		292.50 (194–390)	289 (163–356)	209.0 (176–329) *
C-reactive protein (mg/dL)	0.50 (0.29–0.87)	0.38 (0.10–1.0)	0.81 (0.31–1.10)	0.30 (0.20–1.02)	0.40 (0.20–0.74)
Albumin (g/dL)	3.60 (3–50–3.79)		3.51 (3.42–3.61)	3.62 (3.40–3.70)	3.60 (3.50–3.74)
Dialysis adequacy (KT/V)	1.20 (1.14–1.33)	1.20 (1.10–1.31)	1.30 (1.23–1.43)	1.25 (1.20–1.30) ^	1.30 (1.29–1.40) ^

DBP = diastolic blood pressure, SBP = systolic blood pressure. Statistical significance is shown vs. switch time point where * *p* < 0.05 and ^ *p* < 0.00001. ^§^ at −3 months prior to switch only data for C-reactive protein and KT/V were collected.

**Table 5:** Proportion of patients experiencing side effects over the 12-month study period.

	Follow-Up Period (Months)				
Events Recorded, *n* (%)	−6	−3	0 (Switch)	+3	+6
Hypersensibility	0 (0)	0 (0)	0 (0)	0 (0)	0 (0)
*Adverse events*					
Gastralgia	1 (1.3)	2 (2.6)	0 (0)	1 (1.3)	0 (0)
Dyspepsia	0 (0)	1 (1.3)	0 (0)	0 (0)	0 (0)
Vomiting	0 (0)	1 (1.3)	0 (0)	0 (0)	0 (0)
Total adverse events	1 (1.3)	4 (5.2)	0 (0)	1 (1.3)	0 (0)

## Data Availability

Not applicable.
